# The Relationship of Walking Intensity to Total and Cause-Specific Mortality. Results from the National Walkers’ Health Study

**DOI:** 10.1371/journal.pone.0081098

**Published:** 2013-11-19

**Authors:** Paul T. Williams, Paul D. Thompson

**Affiliations:** 1 Life Sciences Division, Lawrence Berkeley National Laboratory, Berkeley, California, United States of America; 2 Cardiology, Hartford Hospital, Hartford, Connecticut, United States of America; Innsbruck Medical University, Austria

## Abstract

**Purpose:**

Test whether: 1) walking intensity predicts mortality when adjusted for walking energy expenditure, and 2) slow walking pace (≥24-minute mile) identifies subjects at substantially elevated risk for mortality.

**Methods:**

Hazard ratios from Cox proportional survival analyses of all-cause and cause-specific mortality vs. usual walking pace (min/mile) in 7,374 male and 31,607 female recreational walkers. Survival times were left censored for age at entry into the study. Other causes of death were treated as a competing risk for the analyses of cause-specific mortality. All analyses were adjusted for sex, education, baseline smoking, prior heart attack, aspirin use, diet, BMI, and walking energy expenditure. Deaths within one year of baseline were excluded.

**Results:**

The National Death Index identified 1968 deaths during the average 9.4-year mortality surveillance. Each additional minute per mile in walking pace was associated with an increased risk of mortality due to all causes (1.8% increase, P=10^-5^), cardiovascular diseases (2.4% increase, P=0.001, 637 deaths), ischemic heart disease (2.8% increase, P=0.003, 336 deaths), heart failure (6.5% increase, P=0.001, 36 deaths), hypertensive heart disease (6.2% increase, P=0.01, 31 deaths), diabetes (6.3% increase, P=0.004, 32 deaths), and dementia (6.6% increase, P=0.0004, 44 deaths). Those reporting a pace slower than a 24-minute mile were at increased risk for mortality due to all-causes (44.3% increased risk, P=0.0001), cardiovascular diseases (43.9% increased risk, P=0.03), and dementia (5.0-fold increased risk, P=0.0002) even though they satisfied the current exercise recommendations by walking ≥7.5 metabolic equivalent (MET)-hours per week.

**Conclusions:**

The risk for mortality: 1) decreases in association with walking intensity, and 2) increases substantially in association for walking pace ≥24 minute mile (equivalent to <400m during a six-minute walk test) even among subjects who exercise regularly.

## Introduction

Higher doses of physical activity predict greater longevity and lower risks of cardiovascular disease, diabetes, and other conditions; however, the importance of exercise intensity in achieving these health benefits is less clear [1-7]. Intensity is typically measured in terms of metabolic equivalents (METs), where 1 MET is the energy expenditure of sitting at rest (1 MET =3.5 ml O_2_/kg/min) [8-11]. 

Walking is the most commonly performed physical activity in the United States [12]. It is almost always performed at moderate intensity [8]. Walking less than 2.5 mph on a firm surface expends < 3 METs and would be considered light intensity exercise, and includes such activities as walking for social reasons, between transportation and home or work, and with children [8]. Alternatively, very, very brisk walking at ≥4.5 mph and race walking are vigorous (i.e., ≥6 METs) [8]. Cross-sectionally, greater walking intensity is associated with a lower prevalence of type 2 diabetes, hypertension, and hypercholesterolemia [13] when adjusted for distance. Prospectively, the effects of walking intensity on coronary heart disease risk, mortality, and other health outcomes are unclear, in part because higher exercise intensity leads to greater total energy expenditure [3], and because several large prospective studies have yielded conflicting results on whether greater walking intensity predicts lower disease risk when adjusted for total energy expenditure [2,14]. 

A slow walking pace portends a worse prognosis in patients with heart failure, pulmonary hypertension, end stage renal disease, and after coronary artery bypass grafting [15-19]. Patients who achieve less than 400m during a six-minute walk test (i.e., slower than a 24-minute mile) are considered at greatest risk [20,21]. Whether gait can be used to self-identify persons at risk in the general population has not been established. Such a test could have high public health utility given that it requires no special equipment or technical skill to self-administer, and involves no cost.

The National Walkers’ Health Study was established specifically to characterize the dose-response relationships between walking and health outcomes over a broad spectrum of activity levels [13,22-24]. For this reason, the range of walking doses and intensities represented in this cohort are greater than those achievable in geographical, occupational, or gender-based cohorts. This paper uses the 9-year prospective follow-up of this cohort to assess the dose-response relationship between walking intensity (minutes per mile) and the risk of all cause and cause-specific mortality for two important objectives: 1) the potential for prevention, i.e., to assess whether more-intense (faster) walking is associated with lower disease risk when controlling for total amount walked, and 2) screening, i.e., to test whether self-report walking ≥24 minute mile (equivalent to <400m during a six-minute walk test) identifies subjects at substantially elevated disease risk in a non-clinical population, even if they satisfy current physical activity guidelines for health [9]. The associations between walking energy expenditure and cause-specific mortality in this cohort have been reported elsewhere [24].

## Materials and Methods

The National Walkers’ Health Study has been described [13,22-24]. The cohort was recruited primarily between 1998 and 2001 at walking events and from subscription lists to walking publications. Participants completed baseline questionnaires on exercise, height, current and past body weight, body circumferences, diet, current and past cigarette use, and history of disease. Approximately 8% of the 575,000 subscribers solicited elected to join the National Walkers’ Health Study. Our goal was to obtain a sufficiently large cohort for a prospective epidemiologic study of health in walkers rather than a comprehensive survey of these magazine subscribers and walking event participants; thus recruitment among subscribers ceased once over 50,000 questionnaires were received (including multiple surveys from the same individuals).

Walking quantity was taken as the participant’s usual weekly walking distance for the year in which the baseline survey was completed. Walking intensity was the participant’s reply to the survey question, “During your usual walk, how many minutes does it take for you to walk one mile?”. Walking energy expenditure (metabolic equivalent-hours/d or METh/d) was computed by converting the reported distance into duration (i.e., distance/mph) and then calculating the product of the average hours walked per day and the MET value corresponding to their reported pace [8]. Although self-reported walking pace has been shown to be associated with the prevalence of hypertension, high cholesterol, and diabetes cross-sectionally [13], the measurement has not been validated using objectively measured speed in this or other non-patient populations. The study protocol was approved by the University of California Berkeley Committee for the Protection of Human Subjects, and all subjects provided a signed statement of informed consent.

Education was solicited by requesting that the participant provide “years of education (examples: HS=12; BS or BA = 16; MS or MA = 18; PhD or MD = 20).” Height and weight were determined by asking the participant, “What is your current height (in inches, without shoes)?” and, “What is your current weight (pre-pregnancy weight if pregnant)?” Body mass index (BMI) was calculated as weight in kilograms divided by the square of height in meters. Intakes of meat and fruit were based on the questions “During an average week, how many servings of beef, lamb, or pork do you eat”, and “…pieces of fruit do you eat”. Alcohol intake was estimated from the corresponding questions for 4-oz. (112 ml) glasses of wine, 12-oz. (336 ml) bottles of beer, and mixed drinks and liqueurs. Alcohol was computed as 10.8 g per 4-oz glass of wine, 13.2 g per 12 oz. bottle of beer, and 15.1 g per mixed drink. 

Mortality surveillance was completed through December 31, 2008 using the National Death Index (International Classification of Disease codes version 10 [25]). Both underlying and all related deaths (underlying plus entity axis diagnoses of contributing causes) were analyzed. Quartiles of walking speed were defined for the age- and sex-adjusted residuals from multiple regression analyses of sex-specific quadratic polynomials of age. Hazard ratios (HR) from Cox proportional hazard analyses were used to compare all-cause and cause-specific mortality to self-reported minutes per mile walking pace (more minutes = slower walking), with age at death or the end of follow-up as the survival time and age at entry into the study as left-censoring. Covariates for the analyses were sex, years of education, smoking status (yes/no), prior history of a heart attack, aspirin use, walking energy expenditure {METh/d and (METh/d)^2^}, BMI, and intakes of meat, fruit, and alcohol. In these analyses, the competing risk was death due to all other causes [26,27]. Schoenfield residuals were examined for major departures from the proportional hazards assumption. All analyses were performed using the statistical software package Stata (version 11, Stata Corp, College Station, TX). Ninety-five percent confidence intervals (95%CI) are reported for the Hazard ratios. The data are available from the author pending human use approval.

## Results

Of the 9,064 men and 36,427 women surveyed at baseline, 1318 men and 3878 women did not provide their usual walking pace despite providing their usual walking distance, and 323 men and 894 women did not have BMI measurements because of missing height or body weight. Those with these missing data were more likely to be older (mean±SE: 57.52±0.17 vs. 52.37±0.07 years), male (25.6% vs. 19.0%), smokers (8.9% vs. 5.1%), survived a prior heart attack (7.1% vs. 4.0%), less educated (14.41±0.03 vs. 15.19±0.01 years), consume more meat (0.41±0.01 vs. 0.39±0.00 servings/d) and less alcohol (5.82±0.15 vs. 6.43±0.06 g/d) and fruit (1.34±0.01 vs. 1.57±0.01 pieces/d), and walked less (2.39±0.03 vs. 2.94±0.01 km/d) than those with walking pace and BMI (all P<0.0001 with or without adjustment for age and sex). The analyses to follow are restricted to the 7423 men and 31,655 women who had complete data on BMI and walking pace, and whose deaths did not occur within one year of their baseline survey (97 deaths were excluded). Those that were excluded were at 51% greater risk for mortality than those included in the analyses when adjusted for age and sex (HR: 1.51, 95%CI: 1.37 to 1.66, P<0.0001), and 43% greater risk when additionally adjusted for smoking, prior heart attack, education, and intakes of meat, fruit, alcohol, and aspirin (HR: 1.43, 95%CI: 1.30 to 1.57, P<0.0001).

Table 1 presents the cohort’s baseline characteristic by quartiles of the age- and sex-adjusted walking pace. In both sexes, slower adjusted pace (min/mile) was significantly associated with greater BMI, less walking energy expenditure (METh/d), greater meat and less fruit and alcohol consumption. Slower walkers were also more likely to have smoked at baseline. On average, the men were about 10 years older than the women, which contributed to their greater likelihood of having had a previous heart attack at baseline, and greater mortality during follow-up. Simple (unadjusted) all-cause mortality was highest in the slowest walkers. Table 2 presents the baseline characteristics by mortality status.

**Table 1:** Sample characteristics (percent or mean±SD) by walking pace.

	Quartiles of age and sex-adjusted walking pace			
Walking energy expenditure:	1^st^ (fastest)	2^nd^ (penultimate fastest)	3^rd^ (penultimate slowest)	4^th^ (slowest)
**Females**				
Sample (N)	7903	7907	7900	7897
Walking pace (mi per min)*	<13.47	13.47-14.87	14.88-16.80	≥16.81
Walking (METhr/d)¶	2.82±1.83	2.18±1.48	2.10±1.52	1.42±1.29
Age (y)	53.48±13.52	46.97±10.92	49.19±13.15	51.63±12.80
Follow-up (y)¶	9.70±1.28	9.56±1.23	9.62±1.25	9.41±1.13
Education (y)¶	15.04±2.50	15.14±2.48	15.10±2.50	14.81±2.55
Baseline smoker (%)¶	3.71	5.62	5.22	6.53
Ever smoked (%)‡	40.22	38.28	38.38	39.52
Prior heart attack (%)‡	2.54	1.58	1.86	3.08
Meat (serving/d)¶	0.33±0.33	0.38±0.38	0.36±0.37	0.42±0.38
Fruit (pieces/d)¶	1.73±1.16	1.52±1.07	1.61±1.11	1.45±1.07
Alcohol (g/d)¶	6.26±11.29	5.62±10.02	5.67±10.45	4.49±9.59
Aspirin (tablets/d)§	0.27±0.58	0.23±0.54	0.26±0.62	0.30±0.64
BMI (kg/m^2^)¶	24.26±4.07	25.29±4.82	25.24±5.07	28.30±6.69
Mortality (%)§	3.78	1.99	3.03	4.08
**Males**				
Sample (N)	1844	1849	1844	1837
Walking pace (mi per min)*	<13.49	13.49-15.12	15.13-17.05	≥17.06
Walking (METhr/d)¶	2.80±1.89	2.32±1.59	2.08±1.55	1.66±1.37
Age (y)	63.32±13.83	58.84±9.83	60.23±13.75	61.55±12.78
Follow-up (y)¶	9.64±1.21	9.52±1.17	9.52±1.14	9.31±1.05
Education (y)§	15.90±2.69	16.12±2.70	15.89±2.72	15.80±2.80
Smokers (%)‡	3.69	3.84	4.56	5.17
Ever smoked (%)‡	53.85	52.14	52.17	55.85
Prior heart attack (%)‡	12.20	8.87	11.23	13.39
Meat (serving/d)§	0.43±0.40	0.44±0.42	0.46±0.44	0.51±0.47
Fruit (pieces/d)§	1.66±1.26	1.53±1.20	1.52±1.18	1.42±1.13
Alcohol (g/d)§	11.76±17.68	10.43±16.56	9.73±15.42	9.60±16.26
Aspirin (tablets/d) †	0.46±0.73	0.47±0.62	0.45±0.72	0.48±0.86
BMI (kg/m^2^)¶	26.43±3.96	27.16±4.51	27.08±4.52	28.13±5.30
Mortality (%)§	14.75	8.71	13.12	15.08

* Adjusted to the expected value of a 55 year old male. Significant of trend by standard linear regression or logistic regression: † P<0.05; ‡ P<0.01; § P<0.0001; ¶ P<10^-15^

**Table 2:** Sample characteristics (mean±SD) by mortality status.

	Females		Males	
	Dead	Alive	Dead	Alive
Sample (N)	1016	30591	952	6422
Walking (METhr/d)¶	1.72±1.57	2.14±1.62	1.92±1.59	2.26±1.67
Walking pace (min/mi)¶	19.59±6.19	17.20±4.52	19.30±6.01	17.35±4.20
Age (y)¶	66.55±13.73	49.78±12.49	72.51±10.86	59.27±12.11
Education (y)¶	14.50±2.57	15.04±2.50	15.42±2.80	16.00±2.71
Baseline smoker (%)‡	6.89	5.21	5.67	4.11
Ever smoked (%)¶	46.56	38.85	65.44	51.73
Prior heart attack (%)¶	9.35	2.03	24.79	9.44
Meat (serving/d)*	0.39±0.37	0.37±0.37	0.48±0.47	0.46±0.43
Fruit (pieces/d)†	1.71±1.20	1.57±1.11	1.55±1.20	1.53±1.20
Alcohol (g/d)	5.28±11.85	5.52±10.32	10.13±17.25	10.42±16.41
Aspirin (tablets/d)¶	0.55±1.02	0.45±0.69	0.40±0.66	0.26±0.59
BMI (kg/m^2^)	25.87±5.84	25.77±5.45	26.85±4.83	27.25±4.60

Sex-adjusted difference by mortality status significant at : * P<0.05; † P<0.01; ‡ P<0.001; § P<0.0001; ¶ P<10^-15^

### Total all-cause mortality

There were 952 men (12.9%) and 1016 women (3.2%) who died during follow-up. Men and women who smoked at baseline were at 2.5-fold (HR: 2.53, 95%CI: 1.93 to 3.32, P<10^-10^) and 2.1-fold (HR: 2.10, 95% CI: 1.65 to 2.96, P<10^-8^) greater risk for dying than nonsmokers, respectively. A prior heart attack at baseline was associated with 1.6-fold greater risk for mortality in men (HR: 1.60, 95%CI: 1.38- to 1.86-fold, P=10^-9^) and 1.5-fold greater risk in women (HR: 1.53, 95%CI: 1.24 to 1.90, P<0.0001). Greater BMI was also associated with greater mortality in both men (HR: 1.04 per kg/m^2^, 95% CI: 1.02 to 1.05, P=10^-6^) and women (HR: 1.02 per kg/m^2^, 95% CI: 1.01 to 1.03, P=0.02). Other than age and METh/d walked, the only other significant predictor of mortality was aspirin use in women (HR: 1.02 per tablet/d, 95% CI: 1.01 to 1.03, P=0.001).

**Figure 1:**
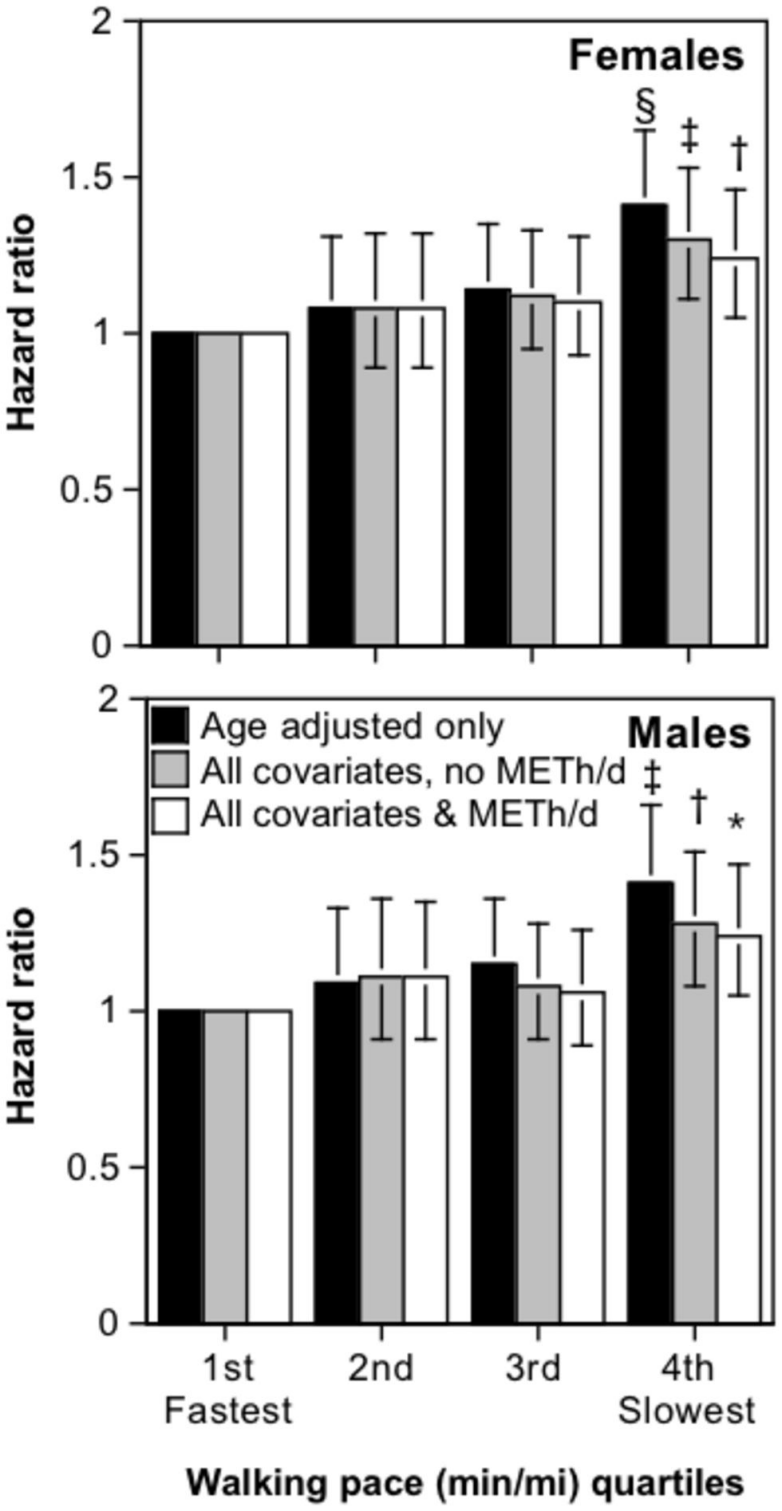
Hazard ratio for all cause mortality by quartiles of walking pace (min/mi, highest quartiles slowest pace) adjusted for age (age and age^2^). Standard covariates include education, smoking status, daily intakes of alcohol, meat and fruit, aspirin use, and prior heart attack. Additional adjustment for walking energy expenditure where indicated. Significance levels for the risk reduction relative to being in the fastest (1^th^) quartile are coded: * P<0.05, † P<0.01, ‡ P<0.001, and § P<0.0001.

Figure 1 shows that a slower walking pace was significantly associated with greater risk for total (i.e., all cause) age-adjusted mortality in both males and females, with the association due primarily to greater mortality in the slowest (4^th^) pace quartile. Adjustment for the other covariates and walking energy expenditure somewhat attenuated the risk increase associated with slower gait. There was little difference in the risk increase per minute/mile walked between men and women (P=0.61 for sex-by-mi/min interaction, P=0.63 for sex-by-slowest quartile of gait interaction), therefore males and females were combined and sex-adjusted. For males and females combined, the risk of total mortality for the slowest (4^th^) quartile of walking pace was 17.6% greater than for faster walkers (1^st^ through 3^rd^ quartiles) when adjusted for walking energy expenditure and other covariates (HR: 1.176, 95%CI: 1.067 to 1.297, P=0.001). A two-standard deviation difference in walking pace was associated with a 17.5% difference in the risk for all-cause mortality (HR: 1.175, 95%CI: 1.093 to 1.262, P=10^-5^) for the sexes combined.

### Cardiovascular diseases

As an underlying cause, Table 3 shows that the relative risk for dying was 2.4% greater per min/mile for all cardiovascular disease (CVD), 2.8% greater for ischemic heart disease, 6.5% greater for heart failure, and 6.2% greater for hypertensive heart disease when adjusted for METh/d walked and other covariates. In contrast, dysrhythmias, cerebrovascular disease, and other CVD as underlying causes of death were not significantly related to walking pace when adjusted for walking energy expenditure and other covariates. The sex-specific increases in risk per min/mile were 2.7% in males (HR: 1.027 per min/mile, 95%CI: 1.009 to 1.047, P=0.004) and 2.0% in females (HR: 1.020 per min/mile, 95%CI: 1.000 to 1.039, P=0.05) for total CVD, 2.9% in males (HR: 1.029 per min/mile, 95%CI: 1.004 to 1.054, P=0.02) and 2.8% in females (HR: 1.028 per min/mile, 95%CI: 1.000 to 1.056, P=0.05) for ischemic heart disease, 6.9% in males (HR: 1.069 per min/mile, 95%CI: 1.026 to 1.113, P=0.001) and 5.5% in females (HR: 1.055 per min/mile, 95%CI: 0.959 to 1.149, P=0.22) for heart failure, and 6.3% in males (HR: 1.063 per min/mile, 95%CI: 1.002 to 1.129, P=0.04) and 6.5% in females (HR: 1.065 per min/mile, 95%CI: 0.996 to 1.140, P=0.07) for hypertensive diseases. The risk increase per min/mi was not significant between men and women for total CVD (P=0.75), ischemic heart disease (P=0.75), heart failure (P=0.64), or hypertensive diseases (P=0.77). This general consistency across genders suggests a robustness to the associations. Table 3 shows that the results for all related mortality (i.e., contributing and underlying causes of death) were generally consistent with those for underlying cause, except that walking pace significantly predicted the risk for the 379 dysrhythmias-related deaths but not the 37 deaths with dysrhythmias as the underlying cause, and the risk for hypertensive disease as an underlying cause but not for all hypertensive disease related deaths. 

**Table 3:** Hazard ratios (95% confidence interval) from proportional hazard analyses for cause-specific mortality vs. min/mile walking pace in 38,981 participants of the National Walkers’ Health Study.

	Deaths		Risk increase per min/mi of walking pace (95% confidence interval)	
	Underlying	All related	Underlying	All related
All cause mortality	1968		1.018	
			(1.007, 1.029)	
			P=10^-5^	
Cardiovascular disease (ICD_10_ I00-I78)	637	1017	1.024	1.019
			(1.010, 1.038)	(1.009, 1.030)
			P=0.001	P=0.0003
Ischemic heart disease (ICD_10_ I20-I25)	336	472	1.028	1.016
			(1.010, 1.047)	(1.000, 1.033)
			P=0.003	P=0.05
Heart Failure (ICD_10_ I50)	36	185	1.065	1.038
			(1.024, 1.107)	(1.017, 1.059)
			P=0.001	P=0.0003
Hypertensive diseases (ICD_10_ I10-I13)	31	207	1.062	1.021
			(1.014, 1.111)	(0.996, 1.046)
			P=0.01	P=0.10
Dysrhythmias (ICD_10_ I46-I49)	37	379	1.014	1.025
			(0.963, 1.068)	(1.008, 1.042)
			P=0.60	P=0.003
Cerebrovascular disease (ICD_10_ I60-I69)	115	194	0.979	1.017
			(0.943, 1.017)	(0.990, 1.044)
			P=0.28	P=0.23
Other cardiovascular disease	82	73	1.025	1.025
			(0.986, 1.066)	(0.984, 1.069)
			P=0.21	P=0.23
Diabetes (ICD_10_ E10-E14)	32	138	1.063	1.017
			(1.019, 1.109)	(0.990, 1.045)
			P=0.004	P=0.21
Dementia (ICD_10_ F01-F06)	44	108	1.066	1.036
			(1.029, 1.104)	(1.006, 1.067)
			P=0.0004	P=0.02

### Secondary prevention in heart attack survivors

Three hundred thirty one of the 1558 subjects who reported a prior heart attack at baseline died during follow-up, including 231 deaths that were CVD-related. Their adjusted risk increased 2.4% per min/mile for all-cause mortality (HR: 1.024 per min/mi, 95%CI: 1.005 to 1.044, P=0.01). The increase in risk did not appear to be specific to CVD-related mortality (HR: 1.018 per min/mi, 95%CI: 0.996 to 1.041, P=0.11 for all CVD-related mortality; and HR: 1.033 per min/mi, 95%CI: 0.996 to 1.070, P=0.08 for all non-CVD-related mortality).

### Mortality in hypertensives and hypercholesterolemics

There were 741 deaths (469 CVD-related deaths) among the 6,566 walkers who reported taking hypertensive medications at baseline, and 427 deaths (265 CVD-related deaths) among 3898 walkers who took cholesterol medications at baseline. The risks for all-cause mortality increased 1.6% (HR: 1.016 per min/mi, 95%CI: 1.003 to 1.028, P=0.02) and all CVD-related mortality increased 1.4% (HR: 1.014 per min/mi, 95%CI: 0.998 to 1.030, P=0.08) per min/mile in the hypertensive medication users. Among those who reported using cholesterol medication at baseline, the risk for all-cause mortality increased 2.1% (HR: 1.021 per min/mi, 95%CI: 1.005 to 1.038, P=0.01) and the risk for all CVD-related mortality increased 2.3% (HR: 1.023 per min/mi, 95%CI: 1.002 to 1.046, P=0.04) per min/mi.

### Diabetes (E10-E14)

The risk for diabetes death increased significantly per min/mile walked for all patients (Table 3), and in patients not using diabetes medication at baseline (HR: 1.065, 95%CI: 1.006 to 1.127, P=0.03 as an underlying cause). 

### Dementia (F01-F06)

The risk of death attributed to dementia increased 6.6% per min/mile, due entirely to the greater risk among the slowest walkers (HR: 2.936 vs. faster walkers, 95%CI: 1.563 to 5.515, P=0.001 for dementia as an underlying cause, and HR: 1.624 vs. faster walkers, 95%CI: 1.075 to 2.454, P=0.02 for all dementia-related deaths). The increased risk persisted when the analyses were restricted to walkers ≥60 years old at baseline (HR: 1.065 per min/mi, 95%CI: 1.027 to 1.104, P=0.0006, same 44 cases). For this older age group, the risk for fatal dementia was 2.91-fold higher among the slowest walkers’ quartile vs. the faster quartiles (HR: 2.911, 95%CI: 1.544- to 5.488-fold, P=0.001) as an underlying cause, and 1.62-fold higher among the slowest walkers’ quartile vs. the faster quartiles (HR: 1.623, 95%CI: 1.063- to 2.477-fold, P=0.03) for all dementia-related deaths.

### Other underlying causes

Walking pace was not significantly related to total or malignant neoplasms (P=0.74), infectious and parasitic diseases (P=0.13), digestive diseases (P=0.19), non-diabetic endocrine diseases (P=0.21), genitourinary diseases (P=0.73), respiratory diseases (P=0.07), diseases of the nervous system (P=0.06), or deaths due to external causes (P=0.31, results not displayed).

### Premature (age<65) mortality

There were 4,414 men who were < 65 years old at baseline, of whom 123 died before the age of 65. Their risk increased 4.14% per min/mi for total premature mortality (HR: 1.041 per min/mi, 95%CI: 1.011 to 1.072, P=0.007). 

### Further exclusion of early mortality

Early mortality did not account for the observed events. When the 339 deaths occurring within the second and third years of follow-up were excluded, Table 4 shows that slower min/mile walking pace remained significantly related to increased risk for all-cause mortality, CVD (underlying and all related), ischemic heart disease (underlying), heart failure (underlying and all-related), hypertensive disease (underlying), dysrhythmia (all-related), diabetes (underlying), and dementia (underlying).

**Table 4:** Effects of excluding deaths occurring within the first three years since baseline on the hazard ratios of all-cause and cause-specific mortality vs. min/mile walking pace in 38,642 participants of the National Walkers’ Health Study.

	Deaths		Risk increase per min/mi of walking pace (95% confidence interval)	
	Underlying	All related	Underlying	All related
All cause mortality	1630		1.018	
			(1.010, 1.027)	
			P=0.0001	
Cardiovascular disease (ICD_10_ I00-I78)	520	843	1.021	1.021
			(1.006, 1.036)	(1.010, 1.032)
			P=0.006	P=0.0002
Ischemic heart disease (ICD_10_ I20-I25)	268	380	1.028	1.016
			(1.007, 1.049)	(0.998, 1.035)
			P=0.008	P=0.09
Heart Failure (ICD_10_ I50)	35	158	1.063	1.042
			(1.027, 1.110)	(1.020, 1.065)
			P=0.001	P=0.0002
Hypertensive diseases (ICD_10_ I10-I13)	26	177	1.068	1.019
			(1.016, 1.123)	(0.994, 1.046)
			P=0.01	P=0.14
Dysrhythmias (ICD_10_ I46-I49)	31	379	1.034	1.030
			(0.989, 1.080)	(1.013, 1.048)
			P=0.14	P=0.001
Cerebrovascular disease (ICD_10_ I60-I69)	93	160	0.956	1.019
			(0.910, 1.005)	(0.990, 1.049)
			P=0.08	P=0.20
Other cardiovascular disease	82	73	0.992	1.005
			(0.948, 1.038)	(0.961, 1.050)
			P=0.74	P=0.84
Diabetes (ICD_10_ E10-E14)	29	118	1.068	1.018
			(1.021, 1.116)	(0.990, 1.046)
			P=0.004	P=0.21
Dementia (ICD_10_ F01-F06)	42	99	1.057	1.029
			(1.018, 1.099)	(0.997, 1.062)
			P=0.004	P=0.08

### Six-minute walking test performance

Table 5 shows that those who walked slower than 24.19 minutes per mile (corresponding to ≤400m during a 6 minute walk test) were at greater risk for all-cause mortality, and deaths due to cardiovascular disease, heart failure, and dementia. They were also at greater risk for all dysrhythmia-related deaths. Even among those walkers who satisfied the current physical activity recommendations by walking ≥7.5 METh/wk, those walking slower than 24.19 minutes per mile were at 44.3% greater risk for all-cause mortality, 43.9% greater risk for CVD as underlying cause and 36.3% greater risk for all CVD- related deaths, 2-fold greater risk for heart failure–related deaths, 53% greater risk for dysrhythmia-related deaths, 5-fold greater risk for dementia as an underlying cause, and 2.7-fold greater risk for all dementia-related deaths. 

**Table 5:** Hazard ratios for walking slower than 24.19 minutes per mile (corresponding to ≤400m during a 6 minute walk test) vs. faster pace in participants of the National Walkers’ Health Study.

	Risk increase per min/mi of walking pace (95% confidence interval)			
	All participants N=38,981		Adequately active* N=27,513	
	Underlying cause	All-related deaths	Underlying cause	All-related deaths
All cause mortality	1.283		1.443	
	(1.130, 1.457)		(1.199, 1.737)	
	P=0.0001		P=0.0001	
Cardiovascular disease	1.310	1.303	1.439	1.363
	(1.054, 1.630)	(1.101, 1.542)	(1.040, 1.991)	(1.059, 1.755)
	P=0.02	P=0.002	P=0.03	P=0.02
Ischemic heart disease	1.318	1.233	1.338	1.188
	(0.974, 1.784)	(0.952, 1.597)	(0.859, 2.082)	(0.799, 1.767)
	P=0.07	P=0.11	P=0.20	P=0.40
Heart Failure	2.722	1.527	1.509	2.007
	(1.344, 5.512)	(1.052, 2.217)	(0.446, 5.106)	(1.190, 3.386)
	P=0.005	P=0.03	P=0.51	P=0.009
Hypertensive diseases	2.072	1.362	4.764	1.580
	(0.869, 4.940)	(0.962, 2.015)	(1.483, 15.304)	(0.921, 2.712)
	P=0.10	P=0.08	P=0.009	P=0.10
Dysrhythmias	1.153	1.522	0.408	1.530
	(0.509, 2.612)	(1.170, 1.986)	(0.057, 2.907)	(1.035, 2.262)
	P=0.73	P=0.002	P=0.37	P=0.03
Cerebrovascular disease	1.282	1.282	1.017	1.467
	(0.888, 1.851)	(0.888, 1.049)	(0.429, 2.410)	(0.814, 2.644)
	P=0.19	P=0.20	P=0.97	P=0.20
Other cardiovascular disease	1.370	1.117	2.408	1.118
	(0.698, 2.687)	(0.538, 2.322)	(1.056, 5.495)	(0.417, 2.997)
	P=0.37	0.77	P=0.04	P=0.83
Diabetes	1.534	1.238	1.874	1.248
	(0.640, 3.677)	(0.799, 1.920)	(0.399, 8.803)	(0.611, 2.549)
	P=0.34	P=0.40	P=0.43	P=0.54
Dementia	3.480	1.761	4.978	2.669
	(1.755, 6.901)	(1.091, 2.842)	(2.147, 11.545)	(1.369, 5.202)
	P=0.0003	P=0.02	P=0.0002	P=0.004

* Adequately active defined as walking ≥7.5 METh/wk, and includes 1203 total deaths. As an underlying cause, there were 368 deaths due to major cardiovascular disease, 202 deaths due to ischemic heart disease, 19 deaths due to heart failure, 14 deaths due to hypertensive disease, 22 deaths due to dysrhythmia, 13 deaths due to diabetes, 72 deaths due to nervous system diseases, and 26 deaths due to dementia. For all related deaths (i.e., underlying and contributing) 591 were major cardiovascular disease related, 274 were ischemic heart disease related, 102 were heart failure related, 123 were hypertensive disease related, 217 were dysrhythmia related, 74 were diabetes related, 106 were nervous system disease related, and 59 were dementia related.

## Discussion

Our 9.4-year follow-up of the National Walkers’ Health Study shows that a slower walking pace was significantly related to greater all-cause mortality, and mortality due to CVD, diabetes, nervous system diseases, and dementia. The increased mortality persisted when deaths during the first three years of follow-up were excluded. The results are consistent with the hypothesis that: 1) for prevention, more-intense (faster) walking may reduce disease risk beyond that achieved by walking distance alone, and 2) for screening, slow gait may identify subjects at substantially elevated disease risk, even if they exercise at recommended levels [9]. 

### Prevention

Most prospective prevention studies have compared the effects of vigorous vs. moderate intense exercise [3]. The Physical Activity Guidelines Advisory Committee Report found greater reductions in mortality for vigorous than for less-intense activity in 10 out of 11 studies, including all four studies that controlled for total energy expenditure [3]. Those studies did not address whether more-intense exercise was associated with lower mortality than moderate exercise when total exercise was held constant. Most also included a variety of exercises and other leisure-time activities, which could confound intensity. Present public health recommendations suggest 150 min/wk moderate-intensity or 75 min/wk vigorous-intensity activity, or some combination that expends similar energy, but our results suggest that the health benefits of walking may be greater for a very brisk pace compared to a slower pace. Particularly important is that a faster walking pace was associated with statistically and clinically significant lower risk for total premature mortality.

### Screening

The 6-minute walk test is widely used clinically to evaluate fitness in cardiovascular and respiratory patients [18,19]. It predicts mortality in patients with heart failure [18] and systolic dysfunction [15], and assesses total exercise capacity and not just cardiovascular performance [20]. In one study, the poorest performing quartile of patients with stable coronary heart disease had 4-fold greater risk of cardiovascular events (heart failure, myocardial infarction, and death) compared to the highest quartile [28]. Another showed that the 6-minute walk test predicted two-thirds greater survival in elderly patients following coronary artery bypass grafting [16].

Our results suggest that a slow walking pace is a risk marker outside the clinical setting, and can be self-administered to evaluate individual risk. Over 40% of adults in the United States walk for leisure [9], and 91% in our survey cohort knew their usual walking pace. Those walking slower than 400 meters in 6 minutes were at substantially greater risk for mortality, including death due to CVD, heart failure, and dementia, independent of their age, sex, METh/d walked, and other factors. Even among subjects who exercised at presently recommended levels, there was substantially greater risk for total-mortality, CVD-mortality, and dementia-related mortality for walking slowly. This finding may reflect the well-established epidemiological association between the lowest quartile of cardiorespiratory fitness and greater total and coronary heart disease mortality [29]. In particular, the 6-minute test has been shown to correlate significantly with cardiorespiratory fitness (VO_2_max) in healthy (r=0.49) [30], renal failure (r=0.51) [17], and heart failure patients (r=0.76) [18]. 

### Other cohorts

The current analyses are unique in their comprehensive analyses of cause-specific mortality, their adjustment for METh/d walked, and their use of a cohort specifically established to assess the dose-response relationship between walking and disease. Other studies of walking intensity have yielded conflicting results, or lacked control for walking energy expenditure. An eight-year follow-up of the Nurses’ Health Study showed that the risk of coronary events was 25% lower for walking 2 to 2.9 mph, and 36% lower for walking ≥3 mph relative to those who walked <2 mph, even when adjusted METh/d walked [2]. Alternatively, the Women’s Health Study reported that time spent walking (P=0.01) but not usual walking pace (P=0.55) was related to reduced mortality when both time and pace were considered simultaneously. These results suggest that there was no benefit to walking faster and no benefit from the increased energy required to walk further in the same amount of time. Other studies either do not adjust energy expenditure for time spent walking [1,31], thereby disregarding the greater energy expenditure required to walk faster, or do not adjust for distance or time walked at all [32-34]. 

### Caveats

In these analyses, vital status was known only from the National Death Index and therefore some subjects who have died are likely to be misclassified as alive. The set of covariates used in the analyses are somewhat restricted and all are measured with error, which will cause their effect to be underestimated. Thus, there likely remains some degree of residual confounding of the results. The ability to exercise harder may be an innate quality of health and not directly related to habitual physical activity. For example, rats repetitively bred for high intrinsic exercise capacity exhibit 350% greater running capacity than rats bred for low running capacity, and the high performing rats have substantially lower visceral fat, blood pressure, fasting glucose and insulin concentrations, and triglyceride and free fatty acid concentrations even when prevented from exercise training [35]. Studies in humans also show that walking pace may reflect innate cardiorespiratory fitness and health status that is independent of regular exercise [29]. Specifically, standardized measurements of usual walking pace over a predefined interval predict lower total, cardiovascular, and cancer mortality in older adults not necessarily engaged in any physical activity [36-39]. The relationship of gait and gait speed to greater risk for mortality from dementia may simply reflect early signs of the disease, although physical activity may reduce the risk of dementia [40]. Similarly, the greater risk for heart failure mortality with slower walking could be a consequence of cardiac insufficiency at an early, preclinical, stage of the disease. We also caution that the assignment of an underlying cause of death by physicians may be problematic [41-43], and dilute associations we reported. Finally, we note that subjects who provided complete data on walking pace and BMI appear to have been healthier than those lacking these data, which may affect the generalizability of the results. 

In conclusion, these analyses are consistent with the hypothesis that intensity affects walking’s health benefits. In addition, slow gait, corresponding to poor performance for the six-minute walking test, could be used to self-diagnose risk and the need for more intense medical intervention, even among regular exercisers meeting presently recommended exercise levels.
